# Special Issue on DNA Replication Stress: Summary of Topics Covered

**DOI:** 10.3390/ijms20122934

**Published:** 2019-06-15

**Authors:** Robert M. Brosh

**Affiliations:** Laboratory of Molecular Gerontology, National Institute on Aging, National Institutes of Health, NIH Biomedical Research Center, 251 Bayview Blvd, Baltimore, MD 21224, USA; broshr@mail.nih.gov

A Special Issue of *International Journal of Molecular Sciences* (IJMS) is dedicated to mechanisms mediated at the molecular and cellular levels to respond to adverse genomic perturbations and DNA replication stress (https://www.mdpi.com/journal/ijms/special_issues/DNA_Replication_Stress). The relevant proteins and processes play paramount roles in nucleic acid transactions to maintain genomic stability and cellular homeostasis. A total of 18 articles are comprised in the series, encompassing a broad range of highly relevant topics in genome biology. These include replication fork dynamics, DNA repair processes, DNA damage signaling and cell cycle control, cancer biology, epigenetics, cellular senescence, neurodegeneration, and aging. Below are highlighting primers for the articles which constitute this recently published IJMS Special Issue.

## 1. DNA Replication Fork Dynamics

Although evidence now strongly supports a role of fork reversal for the protection and timely resumption of DNA synthesis when it stalls under conditions of endogenous or exogenously induced DNA damage, the molecular mechanisms and their regulation are still not fully understood. Building upon their interest in the metabolism of unusual DNA structures that arise during periods of replication stress, Eichman’s lab investigated the bacterial RecG DNA helicase and its mechanistic role in fork reversal [1]. Using a combination of protein structural and biochemical strategies, the authors studied the coordination of the RecG ATPase motor with the fork recognition (wedge) domain. They discovered region-specific movements of RecG’s ATPase domain relative to the wedge domain upon DNA binding. Importantly, these studies unveiled a role of a conserved loop within a previously underappreciated motif known as translocation in RecG (TRG) that plays a crucial role in fork reversal and conformational changes in DNA structure. This work provides a useful model for the analysis of other fork reversal enzymes.

Despite the tremendous advances in characterizing the mechanism of DNA replication in eukaryotic cells, progress made in plants lags compared to yeast or mammalian cells. Kwasniewski et al. set out to study the effects of a chemical mutagen (maleic hydrazide (MH)) or gamma irradiation on DNA replication patterns in chromosome metaphase cells of barley by performing pulse 5-ethynyl-2’-deoxyuridine labeling [2]. Their results suggest that MH exerted a more profound effect on replication than gamma irradiation. This technical approach provides a springboard for future studies to delve into replication perturbances in barley as well other plant species characterized by small chromosomes.

Protein mono- and poly-ubiquitylation as a component of the replication stress response has been a topic of tremendous interest in recent years. Yates and Marechal have carefully reviewed this subject, providing a definitive resource for understanding the roles of ubiquitylation modification machinery that operates at stalled forks to allow optimal fork restart and genome maintenance [3]. The review addresses ubiquitylation targets including the single-stranded DNA binding protein Replication Protein A/RPA, the DNA polymerase processivity clamp PCNA, and Fanconi anemia protein complex FANCD2/I. Also discussed are the reversible ubiquitylation processes that prevail during DNA replication stress.

## 2. Alternate DNA Structures

Difficult-to-replicate sequences pose a unique challenge to the DNA polymerases delegated to deal with noncanonical DNA structures and copy the genome. This is the very topic of a review article from Kristin Eckert’s lab [4]. Specialized DNA polymerases help to cope with such unusual DNA structures, and their regulation plays profound roles during oncogene-induced replication stress. Tsao and Eckert provide a very comprehensive and current assessment of the field that is a useful resource moving forward in this hotly studied area of genome metabolism.

Alternate DNA structures and DNA damage have far-reaching effects on human physiology, including neurodegenerative diseases. This topic is addressed by Konopka and Atkin in the context of amyotrophic lateral sclerosis (ALS), a debilitating progressive neurodegenerative disorder characterized by hexanucleotide repeat expansions [5]. The central role of DNA damage is discussed in the review article, as well as potential therapeutic strategies to treat ALS.

## 3. DNA Repair Proteins and Processes

DNA is considered the quintessential information molecule in genome biology. Therefore, the mechanisms of DNA damage and repair are highly valuable to cellular metabolism. Helena et al. review the DNA repair pathways which exist to protect the genome and preserve cellular homeostasis [6]. The analysis is not only relevant to understanding disease pathogenesis but also diagnosis and therapeutic strategies for combating various cancers.

Dr. Marit Otterlei and colleagues have had a longstanding interest in the in vivo response to DNA damage in human cells. Combining both elegant microscopy and mutation analysis, they report their findings from an investigation of the interaction of the replication processivity clamp PCNA with a translesion synthesis (TLS) DNA polymerase known as REV3L that is implicated in DNA synthesis past ultraviolet light-induced lesions [7]. They discovered that a specialized PCNA interacting motif designated APIM is critical for the function and specificity of REV3L in TLS. Moreover, the study revealed that mutation frequencies and spectra could be modulated in vivo by a PCNA-targeting cell-penetrating peptide, suggesting the potential use of the peptide in chemotherapy strategies to downregulate mutation frequency, as it preferentially targets TLS compared to error-free DNA repair.

Wang et al. characterized the catalytic activity of an archaeal thermophilic endonuclease IV to incise apurinic/apyrimidinic (AP) analogues in single-stranded or double-stranded DNA [8]. Using a battery of AP analogues with different length alkane, polyethylene glycol, cyclic, or two-carbon atom chain spacers, the authors were able to systematically assess substrate specificity and biochemical activity for the recognition and cleavage of phosphodiester bonds by the *Thermococcus eurythermalis* endonuclease IV, providing a model for studies of related enzymes and shedding light on the repair of AP sites in hyperthermophilic archaea.

Human exonuclease I (EXO1) is a DNA processing enzyme with important pleiotropic roles in cellular DNA metabolism. Guido Keijzers et al. review the replication and post-replication functions of EXO1 to help the reader appreciate the involvement of EXO1 mutations in various cancers [9]. Mismatch repair deficiencies caused by molecular defects of EXO1 mutant alleles is associated with multiple cancers. Some of these mutations reside in the nuclease domain, whereas others reside in domains delegated for protein interaction with the mismatch repair factors MLH1 and MSH2. Thus, microsatellite instability driven by EXO1 mutational defects may very well underlie chromosomal destabilization and be a major driver of tumorigenesis. 

Since the discovery over two decades ago that recessive mutations in the RecQ helicase gene *WRN* are linked to the premature aging disorder Werner syndrome, the hereditary disease has served as a window to understanding the molecular basis for genomic stability, yet its precise functions in nucleic acid transactions are still not well understood. The Asaithamby lab addresses the role(s) of WRN in replication fork processing and the post-translational modifications that fine-tune its pathway activities [10]. The authors discuss the proposed dual roles of WRN in replication fork stabilization and pathway choice for double-strand break repair. Interpretations of WRN’s involvement in cellular senescence and genome maintenance place the experimental studies of WRN in a useful perspective for potential clinical implications.

Single-strand breaks are one of the most common DNA lesions in the cell and pose a source of genomic instability by interfering with cellular DNA replication and transcription. A comprehensive review from the Yan lab discusses single-strand break DNA end resection and its step-by-step mechanism [11]. An emphasis is placed on the role of AP endonuclease 2 (APE2) in the process of single-strand break end resection. A valuable perspective for future studies in this area is provided.

## 4. Cell Cycle Control

Understanding how DNA replication initiation is controlled during the DNA synthesis S-phase in mammalian cells is of considerable interest given the number of proteins involved and the importance of ensuring that genome duplication occurs only once per cell cycle. Sokka et al. focused their analysis on the importance of the ATR-activation domain of the activator DNA topoisomerase-2-binding protein 1 (TopBP1) for the suppression of origin firing within the S-phase [12]. By employing DNA fiber assays and human cells expressing a conditionally expressed TopBP1 mutant that is defective in ATR activation, they observed the loss of dormant origin suppression underlying the elevated DNA replication initiation. A model is presented whereby TopBP1 binds to the pre-initiation complex to initiate new forks and activate ATR to inhibit the firing of nearby dormant origins.

A review by Claudio Talora and co-workers addresses the topic of checkpoint adaptation, a process whereby cancer cells acquire mutations in the face of DNA damage and replication stress to survive and continue to proliferate [13]. Although there is much known about DNA damage-induced cell cycle surveillance systems (including checkpoints mediated by CHK1 and CHK2), as well as the sensors, transducers, and mediators involved in the DNA damage response, the molecular mechanisms of checkpoint adaptation are less well understood, particularly in mammalian cells. The key factors in yeast and *Xenopus* are discussed. In addition, the consequences of checkpoint adaptation are described. This review provides a nice perspective of the cellular response to DNA damage stress, placing it in the context of cancer cell survival.

The Bergoglio lab provides a very comprehensive assessment of dormant origins and their role in response to replicative stress to preserve the genome [14]. Origin licensing and firing as well as the spatial and temporal organization of replication origins is discussed. The selection of origins is a complex process that deserves further attention. How dormant origins are affected by replicative stress and the significance of fork speed are active areas of investigation. The mechanisms whereby cells regulate dormant origins and their firing is considered. The functional roles of such proteins as those implicated in Fanconi anemia, Rap1-Interacting Factor, and MCM are described, as are the consequence of deficiencies (e.g., genomic instability) due to loss of these proteins.

## 5. Cancer Biology

Shu-Yan Li and colleagues investigated the basis for variability in the sensitivity of human lung cancer cells as a function of p53 status to the potential anticancer drug 8-chloro-adenosine (8-Cl-Ado) currently in a phase I clinical trial for treatment of chronic lymphocytic leukemia [15]. They determined that p53-null lung cancer cells are hypersensitive to the agent due to elevated double-strand breaks. Their results suggest that several factors play into the heterogeneity of the DNA damage response including defective p53-p21 signaling, poor induction of the DNA repair protein p53R2, and cleavage of the DNA damage sensor PARP-1. In this age of emerging personalized medicine, characterization of the DNA damage response in specific mutant backgrounds of cancer cells may enhance chemotherapeutic strategies.

## 6. DNA Damage and Epigenetics

A review from Sudha Sharma’s lab provides a fresh perspective on oxidative DNA damage in the context of the cellular response and repair mechanisms, as well as the effects of oxidative DNA damage on gene expression [16]. A particularly unique and interesting viewpoint on the epigenetic functions of oxidative DNA lesions, the so-called “stress marks on the genome”, is provided. The preferential occurrence of guanine oxidation in gene promoters may provide a cellular signal to affect the expression of redox-regulated genes. A potential role of G-quadruplexes in this regulation is discussed. Readers are encouraged to read the Sharma paper to acquire new insights into the oxidative stress DNA damage response and the latest developments in this new area of study. 

## 7. Aging, DNA Damage Signaling, and Cellular Senescence

Cellular senescence and its role in aging and neurodegenerative disease is the subject of a comprehensive review contributed jointly by the Gorgoulis and Papadopoulos labs [17]. The counterproductive effects of cellular senescence on chronic inflammation, compromised regenerative capacity, and loss of nerve cell, tissue, and cerebral function are discussed. This informative background provides the authors an opportunity to comment on new and emerging neuroprotection treatment strategies that involve cellular senescence as a therapeutic target.

Stuart Maudsley and colleagues present a review article on the importance of G protein-coupled receptor (GPCR) systems as stress sensors for intracellular damage and as regulators of DNA damage response systems [18]. The various GPCR signaling systems are described systematically and discussed in the context of DNA damage signaling pathways. This leads the authors to propose an emerging field of GPCR therapeutics to regulate DNA damage and repair processes that would in turn influence aging processes.

## 8. Perspective

As Guest Editor for this IJMS Special Issue, I am very pleased to offer the collection of riveting articles centered on the theme of DNA replication stress. The blend of articles builds upon a theme that DNA damage has profound consequences for genomic stability and cellular homeostasis that affect tissue function, disease, cancer, and aging at multiple levels and by unique mechanisms. I thank the authors for their excellent contributions which provide new insight into this fascinating and highly relevant area of genome biology.
